# Effective strategies in ending weight stigma in healthcare

**DOI:** 10.1111/obr.13494

**Published:** 2022-08-07

**Authors:** Britta Talumaa, Adrian Brown, Rachel L. Batterham, Anastasia Z. Kalea

**Affiliations:** ^1^ Division of Medicine University College London London UK; ^2^ Centre for Obesity Research University College London London UK; ^3^ Bariatric Centre for Weight Management and Metabolic Surgery University College London Hospital NHS Trust London UK; ^4^ UCLH Biomedical Research Centre National Institute of Health Research London UK; ^5^ Institute of Cardiovascular Science University College London London UK

**Keywords:** healthcare professionals, obesity stigma, weight bias

## Abstract

Weight stigma impacts negatively healthcare quality and hinders public health goals. The aim of this review was to identify strategies for minimizing weight bias among healthcare professionals and explore future research directions. An electronic search was performed in PubMed, PsycINFO and Scopus (until June 2020). Studies on weight stigma reduction in healthcare students, trainees and professionals were assessed based on specific inclusion and exclusion criteria. A narrative synthesis was undertaken to analyze emerging themes. We identified five stigma reduction strategies in healthcare: (i) increased education, (ii) causal information and controllability, (iii) empathy evoking, (iv) weight‐inclusive approach, and (v) mixed methodology. Weight stigma needs to be addressed early on and continuously throughout healthcare education and practice, by teaching the genetic and socioenvironmental determinants of weight, and explicitly discussing the sources, impact and implications of stigma. There is a need to move away from a solely weight‐centric approach to healthcare to a health‐focused weight‐inclusive one. Assessing the effects of weight stigma in epidemiological research is equally important. The ethical argument and evidence base for the need to reduce weight stigma in healthcare and beyond is strong. Although evidence on long‐term stigma reduction is emerging, precautionary action is needed.

## 1 INTRODUCTION

Social stigma is a fundamental driver of population health inequalities.^1^ Although this has been recognized for decades,^2^ detrimental effects of the stigma of body size and weight have gained wider acknowledgement only recently.^3^ Societal weight stigma is pervasive^4^, ^5^ and suggested to be in part driven by the increased blame and shame framing of obesity in media and public health,^6^, ^7^, ^8^ the cultural reinforcement of a slim ideal^9^ and proclivities for social stratification.^10^ Between 2017 and 2020, weight shaming, a manifestation of weight stigma, decreased slightly in the United States, and although this is promising, blaming individuals with obesity saw little change in the United Kingdom.^11^ However, there is substantive evidence to show that weight stigma is unfair and unjustified, it creates health disparities and hampers healthcare and public health efforts.^3^, ^12^

The stress of stigmatization, from direct experience, but also from stigma suspicion and anticipation, can elicit physiological, psychological and behavioral responses, which harm health over time. Studies show that weight stigma can negatively impact on cortisol, glycated hemoglobin, oxidative stress and C‐reactive protein,^13^ as well as promote global dysregulation of lipid and glucose metabolism, and inflammation.^14^ People with obesity experience weight stigma frequently, almost daily on average.^15^, ^16^ When compared with lower weight counterparts, those with measured or self‐perceived overweight have shown blunted cortisol responses to acute stressful stimuli,^17^ suggestive of sustained elevated cortisol levels. This is consistent with prior research^18^, ^19^, ^20^ showing that although acute stigmatizing stimuli is associated with cortisol reactivity, blunted cortisol responses are more common after persistent and severe chronic exposure to stressors, including weight‐related stigmatization, which often results in feelings of shame.^21^ Although the relationship between adiposity and glucocorticoid dysregulation is complex and several other metabolic and genetic mechanisms have been suggested,^22^ weight stigma has been found to contribute to the interindividual variation in stress response^23^ among people with obesity. Jung et al.^23^ showed that among people with body mass index (BMI) > 30 kg/m^2^, those with low levels of self‐stigma react to acute psychological stress as predicted with an increase in cortisol secretion, whereas those with medium or high self‐stigma show an atypical blunted cortisol response. When obesity was found to predict physiological dysregulation over a 4‐year period, 29% of this effect was explained by weight discrimination alone.^24^ Furthermore, weight stigma is linked to psychological distress, depression, anxiety,^25^ low self‐esteem, and body image disturbances,^13^ often leading to decreased health motivation^16^ and maladaptive coping such as avoidance of timely healthcare, social isolation, reduced physical activity and disordered eating behaviors.^26^ Weight stigma has been shown to increase risk of developing obesity,^8^, ^27^ and it may shorten life‐expectancy, as it is associated with nearly 60% greater mortality risk, not accounted for by traditional physical and psychological risk factors.^28^

Mounting evidence shows associations between weight stigma and increased food intake, eating in the absence of hunger, emotional eating, binge eating and long‐term weight gain.^29^, ^30^, ^31^ Multiple experimental studies have shown that weight discriminatory experiences lead to decreased inhibitory control and increased caloric intake.^32^, ^33^, ^34^ These eating behaviors are likely mediated by emotional distress and dysregulation^35^, ^36^ and should not be considered personal failings but maladaptive coping strategies to unfair treatment.^26^, ^37^, ^38^, ^39^ Furthermore, weight stigma is unique compared with other social stigmas, as prejudices tend to be accepted by people across the weight spectrum.^40^ Internalized weight bias (IWB) encompasses self‐blame and self‐devaluation that results from endorsing negative social messages around weight and applying them to the self.^3^ IWB is believed to explain the relationship between acutely experienced or indirectly perceived weight stigma and maladaptive eating behaviors^41^, ^42^, ^43^ as well as body shame and dissatisfaction, exercise and healthcare behaviors, bodily pain and parental weight talk.^44^ Stigma may lead to efforts of escaping discrimination through weight‐loss attempts,^45^ and thus, some have argued that it may have a positive role in motivating individuals to engage in health behaviors. However, stigmatization creates a dual and countervailing effect of increasing motivation to engage in unhealthy weight‐control behaviors, while simultaneously decreasing the perceived capacity to control weight,^46^ and is consistently linked to adverse health behaviors and decreased long‐term health.^47^, ^48^, ^49^ Moralizing elicits an acute urge to defend one's moral identity, prompting responses that are perhaps visible, but not conducive of health.^50^ Furthermore, while the moralized framing of weight common in healthcare may be done with the intention to motivate a desired behavior, it is most likely to have the opposite effect of disengagement and avoidance of said behavior.^50^ Hunger et al.^38^ have proposed a social identity threat model that elucidates the processes linking weight stigma and the cascade of mechanisms causing the deterioration of physical and psychological health, many of which are bidirectionally linked with eating behaviors.^51^ Additionally, the cyclic obesity/weight‐based stigma (COBWEBS) model by Tomiyama^52^ represents weight stigma as a positive feedback loop perpetuated by stigma‐induced increased cortisol and eating behaviors, which promote weight gain and thus further stigmatization.

Healthcare is one of the most common contexts where weight stigmatization occurs.^39^ Physicians have been reported as the second most common source of weight stigma and discrimination.^39^ Remmert et al.^29^ found that over 70% of US adults enrolling in a weight loss programme report stigmatizing healthcare incidences. Similarly, Puhl et al.^53^ found this proportion to be two thirds among adults in weight management programmes across six different countries. Furthermore, people with obesity are twice as likely to report healthcare discrimination compared with those at lower weight.^54^ Extensive evidence highlights strong weight bias among healthcare professionals (HCP) including physicians, nurses, dietitians, psychologists, kinesiologists, students of these disciplines and even obesity specialists.^55^ HCPs are unlikely to deliberately discriminate against their patients. For example, when measured by the Harvard Implicit Association Test, a validated measure of unconscious weight bias, most medical and nursing students exhibit stronger bias when compared with what they knowingly self‐report.^56^, ^57^ Notwithstanding, underlying negative attitudes can lead to enacted stigma, that is, social cues and behaviors that cause the recipient to feel devalued, disrespected or humiliated. Indeed, the majority of weight stigmatizing healthcare experiences reported by patients are not overt, but subtle.^29^ These may include avoiding eye‐contact or physical touch, providing unsolicited or over‐simplified weight‐loss advice or not having appropriately sized equipment at hand. Biases behind enacted stigma can be *explicit*, referring to conscious beliefs, stereotypes and attitudes, or *implicit*, referring to unconscious and automatic processes. Explicit and implicit bias has been shown to lead to over‐attribution of health problems to weight, less time spent with patients and less patient‐centered, positive affective communication.^58^ Additionally, patients with high IWB report greater healthcare avoidance, increased perceived judgement from doctors, lower frequency of obtaining routine check‐ups, less frequent listening and respect from providers, and lower quality healthcare.^53^ Thus, unchecked weight bias among HCPs as well as IWB among patients potentially undermines successful diagnosis, treatment, and outcome.^59^, ^60^

Identifying widely applicable ways to effectively reduce healthcare related weight stigma is urgently needed. In addition to improving healthcare provision, and the health and well‐being of patients with obesity, healthcare that not only avoids, but actively addresses and reduces IWB may help patients better cope with and reduce the effects of stigma until it is minimized in society. Not surprisingly, while stigmatizing does the opposite,^16^, ^61^ empathetic, non‐stigmatizing weight‐related communication can increase patients' health motivation and intention to comply with health professionals' advice.^62^

A recent joint international consensus statement from leading health authorities has called for the elimination of weight stigma,^3^ a process essential to achieving public health goals globally. Addressing negative biases in the healthcare community will help advocate for a culture and society where the respect, dignity and care afforded to each person is not dependent on their body weight. Stigma reduction interventions are a current research priority.^27^ However, there is a paucity in agreed‐upon, effective and practical strategies to target weight‐related prejudice, which contributes to a lack of strategic anti‐stigma actions. Therefore, the aim of this review was to systematically evaluate current knowledge on strategies for minimizing weight bias in healthcare professionals and to identify future research directions.

## 2 MATERIALS AND METHODS

### 2.1 Literature search

A systematic strategy was used to identify weight stigma studies related to healthcare settings and eating behaviors. An electronic search was performed in three online databases (PubMed, PsycINFO and Scopus) from their inception until June 2020. Combinations of the following keywords were used in all databases: “weight bias,” “weight stigma,” “weight discrimination,” “obesity bias,” “obesity stigma,” “obesity discrimination” or “anti‐fat bias”; and “healthcare” or “quality of care,” or “food choice,” “food intake,” “food behav*,” “dietary intake,” “dietary choice” or “eating behav*”. The search strategy was adapted to each database by using additional subject headings in PubMed and PsycINFO. We included papers written in English language and involving human participants. Bibliographies of all included studies were searched manually to identify any literature not retrieved by the main search.

### 2.2 Study eligibility and selection

**FIGURE 1:**
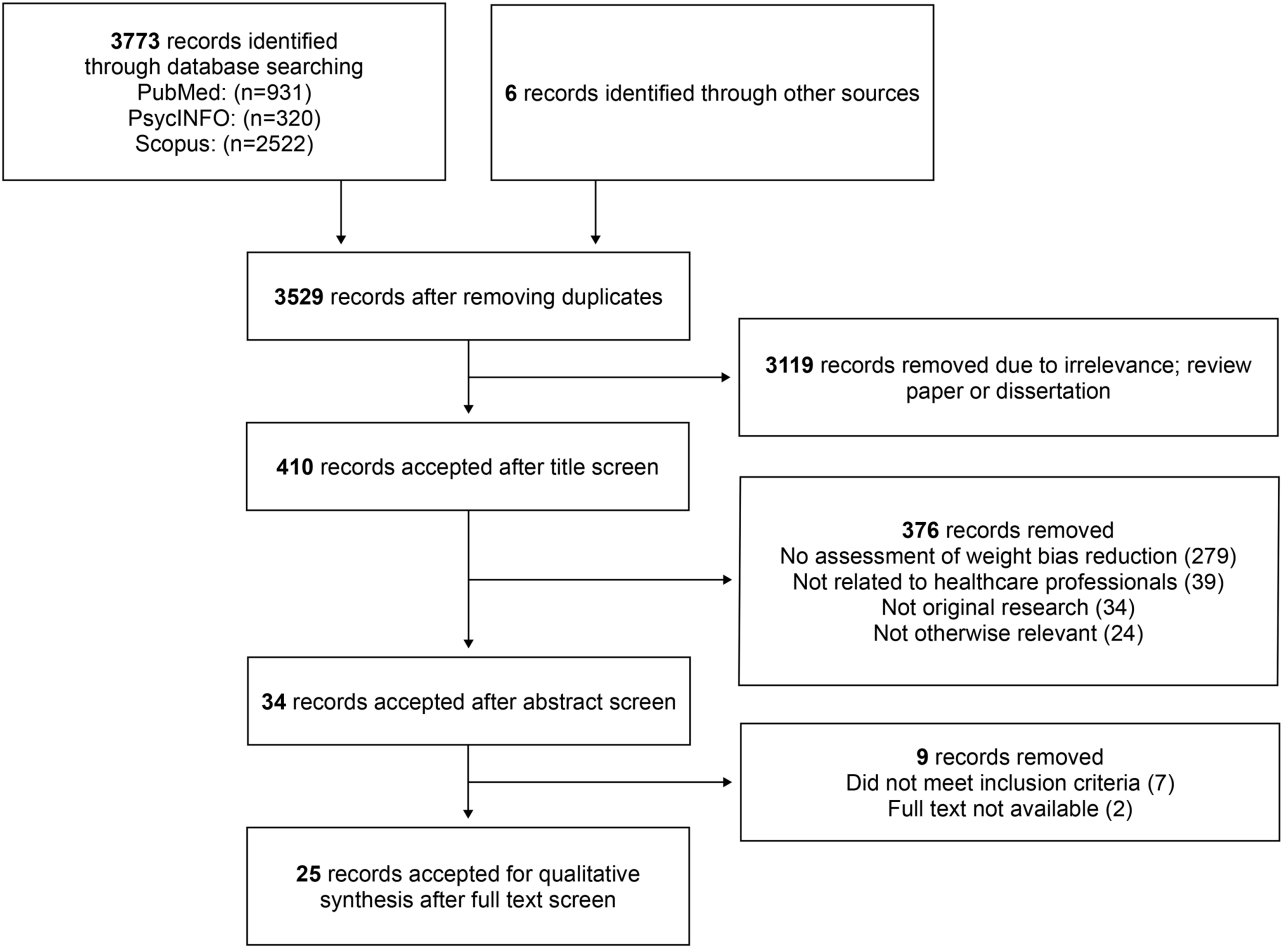
Flow diagram of studies retrieved and included in the scoping review

Studies looking at ways to reduce weight stigma in HCPs were assessed. Inclusion criteria included participants who were healthcare professionals, trainees or students and interventional study designs. Studies were excluded when there was no measurement of effects on stigma. Review papers and observational studies were also excluded. Figure 1 illustrates the selection process in more detail.

### 2.3 Data extraction and synthesis

For all eligible studies, a standardized data extraction table was used to extract data on study authors and year; population and country; intervention type and duration; outcome measures and principal findings. A narrative synthesis was undertaken to identify and analyze emerging themes.

## 3 RESULTS

### 3.1 Key characteristics

The initial search identified a total of 3773 records. A total of 25 interventional studies were included in this review from which seven were randomized controlled trials, four were controlled trials and 14 used a pre‐post intervention design. Three trials included HCPs, two included healthcare trainees, 19 included students, and one trial included both professionals, trainees and students, with a total of 3554 participants across all studies. The duration of studies varied from less than 1 day to 3 years. A summary of studies in healthcare professionals, trainees, and students (Table 1) is presented below. A narrative analysis revealed five major strategies: (i) increased education, (ii) causal information and controllability, (iii) empathy evoking, (iv) weight‐inclusive approach, and (v) mixed methodology. Results are presented below according to these strategies.

**TABLE 1:** Summary of studies in healthcare professionals, trainees, and students

Study	Population	Design	Intervention type and duration	Outcome measure	Main findings
*Increased education*					
Barra and Singh Hernandez (2018)	Nursing students (*n* = 103), USA	Pre‐post intervention	Weekly **obesity sensitivity training** (15 week) on weight‐based discrimination	Newly constructed 5‐item, 4‐point Likert scale questionnaire	**Attitudes improved** on all five items of the questionnaire
Gayer et al. (2017)	Medical students (*n* = 718), USA	Controlled trial	Standard **obesity curriculum** (8–10 h of lectures, virtual patient case presentations)	FPS	**Stereotyping decreased** (FPS: 3.65 vs. 3.37) and remained significant at 3‐year follow‐up
Geller et al. (2018)	Medical students (*n* = 59), USA	Pre‐post intervention	**Ethics seminar** within “Obesity, Nutrition, and Behaviour Change” course discussing personal experiences and weight bias norms	IAT, survey	74% had high implicit bias; 4 months post‐intervention, **attitudes improved in 30%**, did not change in 53%, and worsened in 10%
Jones et al. (2019)	Physiotherapists (*n* = 27), Canada	Pre‐post intervention	**Seminar (8 h)** on obesity and osteoarthritis presented by respected opinion leaders	ATOP, BAOP	**Beliefs improved** (BAOP: 7.4 vs. 22.3), but **attitudes** towards people with obesity **worsened** (ATOP: 71.3 vs. 63.5)
Nickel et al. (2019)	Physicians, nurses, medical students and nursing trainees (*n* = 702), Germany	Randomized controlled trial	**Video teaching (2.5 min)** on obesity disease burden and treatment	FPS	**Stereotyping did not change** when compared with control group in physicians (FPS: 3.5 vs. 3.5), nurses (FPS: 3.3 vs. 3.3), medical students (FPS: 3.5 vs. 3.6) nor nursing students (FPS: 3.4 vs. 3.4)
*Causal information and controllability*					
Brochu (2020)	Psychology trainees (*n* = 45), USA	Pre‐post intervention	**Seminar (3 h)** on weight controllability and weight bias, informed by attribution‐value model of prejudice and HAES	AFA‐willpower, AFA‐dislike, attitudes toward fat clients	1‐week post‐intervention, weight controllability **beliefs** (AFA‐willpower: 4.46 vs. 3.39) and **attitudes** (AFA‐dislike: 2.36 vs. 2.10) **improved**
Diedrichs and Barlow (2011)	Psychology students (*n* = 85), Australia	Controlled trial	**Lecture (2 h)** on behavioral, or multiple causes of obesity and weight bias, informed by HAES	AFAT	**Beliefs and attitudes improved** and remained significant at 3‐week follow‐up (AFAT: 2.18 vs. 2.04 vs. 2.00)
O'Brien et al. (2010)	Health promotion students (*n* = 159), USA	Randomized controlled trial	Weekly **tutorials (3 week)** on genetic/socioenvironmental causes of obesity, oral presentation, and written assignment	AFA, BAOP, pen and pencil version of IAT	**Beliefs worsened** (AFA‐willpower: 4.4 vs. 5.1) **and improved** (BAOP: 23.8 vs. 20.5), and **attitudes** (AFA‐dislike: 2.1 vs. 1.7) and **implicit bias improved** (IAT‐good/bad: 14.2 vs. 10.3, IAT‐lazy/motivated: 11.0 vs. 9.7) in gene/environment group; implicit bias worsened in diet/exercise group (IAT‐good/bad: 14.0 vs. 14.4, IAT‐lazy/motivated: 10.3 vs. 13.1)
Persky et al. (2010)	Medical students (*n* = 110), USA	Randomized controlled trial	**Reading** short article, **clinical encounter** via immersive virtual environment	Newly constructed single‐item questionnaire, OPTS	**Beliefs improved** and **stereotyping decreased** in genetics group compared with control (OPTS: 3.55 vs. 3.69); stereotyping did not change in behavioral group (OPTS: 3.75) compared with genetic and control groups
*Empathy evoking*					
Cotugna et al. (2010)	Dietetics students (*n* = 40), USA	Pre‐post intervention	Following a **calorie‐restricted diet (1 week)**	FPS, newly constructed questionnaire, journal entries	**Stereotyping decreased** (t = 3.184, p < 0.05) significantly
Harris et al. (1991)	Psychology students (*n* = 244), USA	Randomized controlled trial	**Reading** high status or empathy evoking interview with person with obesity together with or without interview with obesity expert	Newly constructed 18‐item, 7‐point Likert scale questionnaire	**No changes in attitudes** were seen in any of the groups
Hunter et al. (2018)	Nursing students (*n* = 29), UK	Pre‐post intervention	Wearing a **bariatric empathy suit (30 min)**	NATOOPS, focus group	Some **attitudes** (NATOOPS‐1: 25 vs. 33, NATOOPS‐2: 64 vs. 82, NATOOPS‐5: 56 vs. 64) **improved**, but **beliefs** and stereotyping did not **change**
Kushner et al. (2014)	Medical students (*n* = 127), USA	Pre‐post intervention	**Reading** and reviewing two weight stigma articles, **clinical encounter (8 min)** with standardized patients with overweight, case observations	Newly constructed questionnaire	**Empathy increased in 48%** and decreased in 23%; **stereotyping decreased in 53%** and **increased in 33%**; at 1‐year follow‐up, empathy remained significant, but stereotyping regressed to back to the baseline mean
Matharu et al. (2014)	Medical students (*n* = 129), USA	Randomized controlled trial	**Dramatic reading (1 h)** of a play script titled “The Most Massive Woman Wins,” nondirective group discussion	AFA, IAT, JSPE, two open‐ended questions	**Empathy increased** in both groups; **attitudes improved** only in intervention group (AFA: 42.6 vs. 38.1); **implicit bias did not improve** (IAT: 0.44 vs. 0.38)
Molloy et al. (2016)	Nursing students (*n* = 70), USA	Pre‐post intervention	Bariatric sensitivity training (1 h) using **6 trigger films (<4 min)** with facilitated group debriefing	BAOP, NATOOPS	**Beliefs** (BAOP: 16.4 vs. 19.0) and some **attitudes** (NATOOPS‐2: 543 vs. 649, NATOOPS‐3: 515 vs. 452, NATOOPS‐4: 66 vs. 61) **improved**; at 30‐day follow‐up, beliefs remained significant (BAOP: 18.2), some attitudes (NATOOPS‐1: 571) reverted back
*Weight‐inclusive approach*					
McVey et al. (2013)	Health promoters (*n* = 325), Canada	Pre‐post intervention	**Interactive workshop** on weight bias, intuitive eating, weight‐centric health messaging and mental health promotion, informed by HAES	AFA, SATAQ, semi‐structured interview	**Attitudes** (AFA: 33.8 vs. 23.99 vs. 25.18) and internalization of sociocultural **stereotypes** (SATAQ: 13.61 vs. 11.48 vs. 12.28) **improved** and remained significant at 6‐week follow‐up
Werkhoven (2020)	Healthcare students (*n* = 124), Australia	Pre‐post intervention	Weekly **lectures and tutorials (12 week)** focusing on nutrition and stigma reduction, informed by HAES	AFA, FSQ, focus group	**Attitudes improved** (AFA: 47.0 vs. 43.1); **stereotyping decreased** (FSQ: −0.32 vs. − 0.24, p > 0.05)
*Mixed methodology*					
Falker et al. (2011)	Health professionals (*n* = 30), USA	Pre‐post intervention	Self‐learning **bariatric sensitivity training** (44 pg) aiming to evoke empathy, addressing multiple causes of obesity and weight stigma	Newly constructed survey	Self‐reported **attitudes** towards patients with obesity **improved**
Luig et al. (2020)	Medical residents (*n* = 32), Canada	Pre‐post intervention	**Lectures** with **bariatric empathy** suit experience, clinical encounter, narrative reflections	ATOP, BAOP, reflective writing	**Beliefs improved** (BAOP: 19.86 vs. 24.03), but **attitudes did not improve** (ATOP: 73.15 vs. 69.26)
Poustchi et al. (2013)	Medical students (*n* = 64), USA	Pre‐post intervention	**Video (17 min)** titled “Weight Bias in Health Care,” interactive discussion	ATOP, BAOP, FPS	**Beliefs** (BAOP: 16.53 vs. 19.27) **and stereotyping** (FPS: 3.65 vs. 3.45) **improved**; **attitudes did not improve** (ATOP: 66.14 vs. 64.90)
Swift et al. (2013)	Nutrition students (*n* = 43), UK	Randomized controlled trial	**Two videos (34 min)** titled “Weight Prejudice: Myths and Facts” and “Weight Bias in Healthcare”	Willpower and dislike subscales of AFA, BAOP, FPS, IAT	**Beliefs** (BAOP: 11.2 vs. 19.9, AFA‐willpower: 5.42 vs. 3.88), **attitudes** (AFA‐dislike: 1.86 vs. 1.45) and **stereotyping** (FPS: 3.7 vs. 3.2) **improved**; **implicit bias did not change** (IAT‐good/bad: 3.8 vs. 2.7, IAT‐lazy/motivated: 4.5 vs. 2.6); at 6‐week follow‐up, changes in beliefs remained significant (BAOP: 13.7, AFA‐willpower: 4.63), but attitudes (AFA‐dislike: 1.57) and stereotyping (FPS: 3.6) did not
Rukavina et al. (2008)	Kinesiology students (*n* = 69), USA	Pre‐post intervention	**Multicomponent intervention** including lecture (75 min), group activity and service‐learning project	AFAT, ERT	**Beliefs** improved (AFAT‐blame: 3.2 vs. 3.41, AFAT‐physical: 3.17 vs. 3.21, AFAT‐social: 4.1 vs. 4.1), but **attitudes** and **stereotyping did not change**
Rukavina et al. (2010)	Kinesiology students (*n* = 78), USA	Controlled trial	**Multicomponent intervention** including interactive discussions, audio tape listening, perspective taking, role‐playing and service‐learning project	AFAT, IAT, ERT	**Beliefs** about controllability (AFAT‐blame 2.88 vs. 2.59) and social value (AFAT‐social: 2.04 vs. 1.97) **improved**; **attitudes**, **stereotyping** and **implicit bias did not change**
Wiese et al. (1992)	Medical students (*n* = 75), USA	Randomized controlled trial	**Seminar (2 h)** including video, reading article about genetic causes of obesity, role‐playing, reflective writing	Newly constructed questionnaire	**Beliefs, attitudes and stereotyping improved**; at 1‐year follow‐up, changes in beliefs and stereotyping remained significant
Wijayatunga et al. (2019)	Kinesiology students (*n* = 67), USA	Controlled trial	**Lecture (80 min)**, **video**, empathy‐evoking group activities and reflective writing	AFAT, IAT	**Beliefs improved** and remained significant at 4‐week follow‐up (AFAT‐blame: 2.79 vs. 2.43 vs. 2.40, AFAT‐physical: 2.59 vs. 2.63 vs. 2.53, AFAT‐social: 1.72 vs. 1.77 vs. 1.76); **implicit bias did not change** significantly (0.55 vs. 0.91)

Abbreviations: AFA, Anti‐Fat Attitudes Questionnaire; AFAT, Anti‐Fat Attitudes Test; ATOP, Attitude Towards Obese Persons; BAOP, Beliefs About Obese Persons; ERT, Explicit Rating Test; FPS, Fat Phobia Scale; FSQ, Fat Stereotypes Questionnaire; HAES, Health at Every Size; IAT, Implicit Attitude Test; JSPE, Jefferson Scale of Physician Empathy; NATOOPS, Nurses Attitudes Towards Obesity and Obese Patients Scale; OPTS, Obese Persons Trait Survey; SATAQ, Sociocultural Attitudes Towards Appearance Questionnaire.

### 3.2 Reducing weight stigma among healthcare professionals and students

#### 3.2.1 Increased education

Literature on medical education suggests that HCPs feel inadequate in caring for patients with obesity.^63^ Negative attitudes towards people with overweight or obesity may be shaped by experiences with poor treatment success, patient non‐adherence and poor long‐term outcome data that result from inadequate knowledge and skills. A possible solution has been to develop educational programmes on obesity and weight‐related health.^64^ Five studies used strategies based upon increasing knowledge of obesity in both students^65^, ^66^, ^67^ and HCPs.^68^, ^69^

The first study used a brief 2.5‐min video that focused on the etiology and treatment of obesity and showed no influence on weight bias in medical students, nurse trainees, nurses or physicians.^69^ In contrast, a comprehensive obesity curriculum delivered to medical students over 3 years did show small, but significant reductions in bias as assessed by the Fat‐Phobia Scale questionnaire; this was maintained at 1‐year follow‐up.^66^ However, resulting values still indicated moderate amounts of weight bias and the clinical relevance of this change is unclear.

Three of the educational studies highlighted a social influence component,^65^, ^67^, ^68^ which is the idea that social factors have a strong impact on people's beliefs and attitudes.^70^ Accordingly, Jones et al.^68^ trialed an 8‐h educational seminar on obesity and osteoarthritis among physiotherapists. The seminar was delivered by respected obesity experts and opinion leaders and the content centered around the complex causes and complications of obesity. Although results showed moderate improvements in beliefs about weight controllability, negative attitudes increased after the seminar. The authors noted that seminar discussions tended to focus on practical and safety considerations, which might have contributed to worsening of attitudes by emphasizing the difficulties of working with patients with obesity. Geller et al.^67^ studied the impact of an ethics seminar embedded within a standard obesity, nutrition and behavior change course. The session revolved around group discussions on the personal experiences (74% struggled with their weight) and social norms (70% showed a thin‐preference on the Implicit Attitudes Test among students). Four months after course completion, 30% of students self‐reported improved attitudes, 53% reported no change and 10% reported more negative attitudes. Barra and Singh Hernandez^65^ trialed a 15‐week medical practicum and obesity sensitivity training in nursing students. The education was supported by weekly exchange of ideas on weight bias and its effects on patient care, with results showing a decrease in negative attitudes and many students articulated awareness and remorse regarding their bias. Based on Lewin's theory of planned change,^71^ the intervention involved the identification and correction of old behaviors and the planning and executing of new ones, aiming to establish a new status quo, which was later observed in students' improved communication style.

Cross‐sectional data suggest that increased general education and a deeper understanding of obesity alone is likely to be insufficient for reducing weight stigma^72^ and in contrast, bias may actually increase as a result.^73^ This may be due to the enforcement of and further socialization to weight stigma norms that are commonly expressed in health‐related education and working environments.^74^ The degree of social influence is dependent on the clarity of social norms expressed by group members,^70^ and thus, even a single person, such as faculty role models,^75^ can have immediate and long‐term effects on student's views and opinions on obesity. The implicit and explicit communication of social norms in educational interventions warrants careful attention, given the possibility of changing bias in both directions. Another possible explanation for the lack of stigma reduction through increased knowledge relates to the content of standard obesity education, which tends to discuss weight from an individualized and medicalized perspective.^76^ Indeed, focusing on individual behaviors as drivers of and as solutions to weight‐related health has been show to increase implicit bias in previous studies.^77^ Therefore, health education needs improvement perhaps not only through the introduction of broader uncontrollable determinants of weight, but also through discussions of the harm caused by social and cultural norms and messages concerning body weight. Opportunities to practice non‐stigmatizing care throughout the education may further support this aim. This may be achieved with inclusive imagery and medical instruments as well as positive patient interactions.

#### 3.2.2 Causal information and controllability

In line with attribution theory, weight bias is arguably shaped by beliefs about the controllability, and thus responsibility, over body weight. Implying that weight is under an individual's control elicits blame, giving justifications to stigmatizing beliefs and stereotypes. Although attributions of behavioral causes of obesity have been shown to predict stronger weight bias,^77^, ^78^ genetic determinism has been shown to decrease bias.^79^ As such, investigators have looked to reduce stigma by changing beliefs about an individual's control over their body weight. Four trials in healthcare students investigated the effects of providing causal information, focusing on the genetic and/or socioenvironmental determinants of weight.^80^, ^81^, ^82^, ^83^

Reading about the genetic determinants of body weight before a virtual clinical encounter led to reduced controllability beliefs when compared with reading about behavioral determinants, or reading nothing.^83^ Negative stereotyping reduced in the genetic condition when compared with control, but not when compared with the behavioral condition. However, students who received genetic information gave patients less health screening advice, possibly indicating the need for filling in the gap in health communication knowledge when weight‐loss advice is not the central focus to care. Diedrichs and Barlow^81^ investigated the effects of 2‐h lectures, which focused either on the multiple determinants or only behavioral determinants of obesity. The multiple determinants lecture involved a detailed study of the empirical evidence demonstrating the multifactorial and uncontrollable nature of weight, as well as practical strategies for avoiding weight stigmatization and promoting Health at Every Size (HAES) principles with patients and in research. Post‐intervention and at 3‐week follow‐up, students who received the multiple determinants lecture showed fewer negative beliefs and attitudes towards people with obesity. The behavioral determinants lecture, following standard curriculum and aiming to increase knowledge about risk factors and treatments of obesity, did not reduce students' beliefs nor attitudes. Notably, the control group, who received no lecture, showed significant increase in social disparagement of people with obesity at both timepoints. However, this should be interpreted with caution as baseline levels of disparagement were significantly lower in this group and the increase in bias may reflect regression to the mean. In another study, three weekly tutorials that presented research on the uncontrollable determinants of weight were successful in improving measures of explicit and implicit attitudes, whereas tutorials focusing on behavioral determinants showed an increase in negative implicit attitudes.^82^ However, although beliefs about controllability decreased when presenting genetic/environmental information, this was not found to be a mediating factor for attitudes in this study. Although post hoc tests revealed a decrease in the dislike subscale of anti‐fat attitudes in the genes/environment group, scores in the willpower subscale, indicating attribution of blame, increased. This, the authors explain, might have been due to regression to the mean, but could also reflect stable beliefs that people with obesity should lose weight regardless of the level of controllability and thus they must require high levels of willpower to overcome any barriers.

When teaching from a weight‐centric perspective, causality interventions may unwittingly reinforce negative values towards higher weight. The attribution‐value model of prejudice adds that social bias increases when, in addition to responsibility, the stigmatized characteristic is negatively valued.^84^ This model was applied by Brochu^80^ who investigated the effects of a 3‐h seminar, addressing weight controllability beliefs, plus negative attitudes and size acceptance. There was a strong emphasis on social justice, decreasing the negative value of overweight and obesity and encouraging a weight‐inclusive paradigm. Measured 1‐week post‐intervention, the seminar was successful in reducing dislike and negative attitudes toward people with obesity. Mediation analysis showed that the reduction in weight controllability beliefs explained these results. However, due to absence of control group, these results should be considered preliminary.

Although studies manipulating controllability beliefs in the general population have yielded mixed efficacy in reducing stigma,^85^ studies in healthcare populations are encouraging. This may be a reflection of the science literacy of this population. Previous studies have found that people in the general population may not believe briefly presented genetic information about obesity.^77^ Even a third of medical students in the genetics group in the study by Persky and Eccelston^83^ showed “mis‐match” responses. Although it is not clear as to why this was the case, it might reflect strongly held beliefs that weight is a personal responsibility pertinent to the clarity, persuasiveness and depth of the information being presented. However, healthcare students and professionals may be more willing to change their views on weight controllability if new information is delivered together with weight‐inclusive health promotion strategies that may improve health regardless of weight status. Thus, when aiming to educate individuals on weight controllability, it is imperative that the information is clear and convincing and delivered in depth.

#### 3.2.3 Empathy evoking

Empathy, the ability to understand the lived experience of another and to communicate that understanding,^86^ is essential to effective therapeutic care.^87^ Empathy‐evoking interventions aim to change attitudes and reduce weight stigma by increasing acceptance and liking of individuals with obesity.^88^ Additionally, limited evidence suggests that in general, weight‐biased attitudes are more easily influenced compared with weight‐biased beliefs.^88^ Although beliefs are often rooted in various personal experiences, available information and knowledge, and are measured via questions on the causes of body weight, attitudes arise from core values and feelings, and are measured via questions on what other people are like, what they can do and how they should be treated. Six studies have investigated the effects of evoking empathy as a strategy to reduce weight stigma.^89^, ^90^, ^91^, ^92^, ^93^, ^94^

Reading about weight stigma, followed by a brief 8‐min interaction with a patient led to significant improvements in empathy and stereotyping in medical students.^92^ However, at 1‐year follow‐up, while empathy remained, negative stereotyping reverted back to the baseline mean. In another study where participants read an interview with a person with obesity, designed to evoke either status or empathy and coupled with or without an interview with an obesity expert, no significant changes occurred in any condition.^90^ Reading the expert interview did increase participants' knowledge of obesity, but this did not contribute to changes in attitudes.

Molloy et al.^94^ showed nursing students six 4‐min videos, depicting stigmatizing and emotive patient scenarios. Beliefs and negative attitudes decreased significantly post‐intervention. However, at 30‐days follow‐up, attitudes regarding negative feelings towards, the characteristics of, and supportive roles in caring for patients with obesity reverted back to the baseline mean. Furthermore, although weight‐biased beliefs in this cohort improved and changes remained significant after follow‐up, scores were still reflective of unacceptable levels of bias as assessed by the Beliefs About Obese Persons questionnaire. Similarly, in another study in nursing students, wearing a bariatric empathy suit for 30‐min reduced negative attitudes in three of five weight bias measurement domains.^91^ In a post‐intervention focus group, students described the experience as “hard,” “feeling trapped,” and “embarrassing,” and later recognized that caring for patients with obesity can be “emotionally draining” and “stressful.” No follow‐up was done, and how this impacted weight stigma in the long‐term is unknown. Furthermore, others have argued that if empathy‐building is not possible without wearing a costume to assume a stigmatized identity, then doing so will be unlikely to be meaningful^95^ because there is a notable proportion of individuals in whom empathy‐building interventions produce paradoxical effects.^85^ People who respond better to this method on the other hand will be able to see that effect using less debatable methods.

Matharu et al.^93^ used dramatic play reading to promote active empathic engagement in medical students. This was compared with a standard 1‐h lecture on the medical management of obesity. Empathy increased in both groups similarly, but attitudes improved significantly only in the theatre group, whereas implicit bias remained unchanged in both. Given that empathy increased similarly in both groups, it is possible that the decreased reporting on explicit attitudes was influenced by social desirability because the discriminatory nature of weight bias was made salient only in the theatre group. Moreover, after 4 months, a follow‐up survey revealed that students in the theatre group did not show increased recognition of weight stigma as something that needed to be addressed in society. However, they were more likely to take a patient‐centered approach to obesity care, whereas students receiving the standard lecture were more likely to take a prescriptive approach and recommend weight loss and exercise. Finally, to increase reflective skills and reduce healthcare students' negative attitudes, Cotugna et al.^89^ had dietetic and health promotion students follow a 1 week 1200–1500 kcal diet, commonly prescribed in weight management. In journal comments, students expressed their difficulty dealing with hunger and social events with 35% not being able to successfully restrict their calories. Limited post‐intervention results showed self‐reported increases in empathy and a significant reduction in negative stereotyping.

The utility and long‐term effects of empathy‐evoking interventions in healthcare populations is unclear. Research shows that empathy does not necessarily decrease weigh stigma.^85^, ^96^ Daníelsdóttir et al.^85^ have argued that evoking empathy is ineffective when it emphasizes the negative aspects of obesity, which reinforces stereotypes of helplessness and weakness. They suggested that rather than eliciting pity, a more constructive aim might be to emphasize size acceptance, respect and the human right for dignity. Size acceptance may be an effective strategy for both HCPs and patients, as accepting one's size, including when living with obesity, has shown to reduce stigma in others.^97^

#### 3.2.4 Weight‐inclusive approach

Concerns over the long‐term ineffectiveness^98^, ^99^ and unintentional negative consequences,^100^, ^101^ including stigmatization, of the dominant weight‐centric health paradigm have drawn research attention to weight‐inclusive approaches.^102^, ^103^, ^104^ The weight‐inclusive approach differs from other approaches in this review by not operating from a priori assumptions that weight loss is achievable, beneficial, safe and necessary for everyone with higher weight. Two interventions^105^, ^106^ focused on a weight‐inclusive approach to health and combined this with weight bias awareness.

A full‐day interactive professional development workshop decreased anti‐fat attitudes and internalized weight stereotypes in health practitioners, which remained significant at 6‐week follow‐up.^105^ The workshop discussed the potential downfalls of weight‐centric healthcare, and taught principles of intuitive eating and mental health promotion. The educational component was supported by salience of stigma reduction goals e.g. one activity included writing weight‐based stereotypes on paper and later tearing it up, symbolizing awareness and rejection of such beliefs. In a post‐intervention survey, the participants described the workshop materials as “credible, current, and evidence‐based.” Although still significant, results at follow‐up started to drift, suggesting the need for continuing long‐term support. This was voiced by the participants in follow‐up interviews as well. Werkhoven^106^ investigated an undergraduate nutrition elective taught from a HAES perspective. A 12‐week curriculum with 3‐h of lectures, tutorials and practical group activities each week led to a strong increase in nutrition knowledge and a moderate decrease in all measured domains of anti‐fat attitudes as assessed by the Anti‐fat Attitudes Questionnaire.^106^ Stereotyping as assessed by the Fat Stereotypes Questionnaire also decreased, but the result did not reach significance. For both measures, post‐intervention levels reflected a low degree of weight bias.

One way in which adopting a weight‐inclusive paradigm may reduce weight bias is by framing obesity not only as a medical and public health issue, but a human rights issue, emphasizing that all individuals, regardless of weight status, deserve dignity and have the right to equal quality healthcare.^107^ Another way is by advocating for size diversity and acceptance,^97^, ^108^ supported by research demonstrating that health promotion is not necessarily dependent on weight loss.^109^, ^110^ Focusing on modifiable health behaviors rather than weight may help build better provider‐patient relationships by empowering both parties. Although results from current studies are promising, conclusions are limited by absence of controlled studies and further research is needed.

#### 3.2.5 Mixed methodology

It may be that due to the complex nature of bias formation, a combination of established as well as other strategies are required. Eight interventions utilized a mix of various methods, mostly causal information, empathy evoking and stigma awareness raising. With most interventions being delivered in lecture or tutorial format, with or without additional video, reflective writing or role‐play components.

Two interventions combined general education and causal information.^111^, ^112^ A self‐learning online module, designed to address the multiple causes of obesity and increase awareness of weight stigma, was shown to decrease the likelihood of stigmatizing attitudes in HCPs 1‐month after completion.^111^ However, results were based on participants' subjective evaluation of their awareness and attitudes of obesity, bearing risk of social desirability bias. Luig et al.^112^ assessed a pilot course aiming to improve family medicine residents' knowledge and confidence with obesity counselling. The program focused on general knowledge and counselling skills, but combined lectures with a bariatric empathy suit experience and reflective writing. Weight stigma was not explicitly addressed in the course. Although the residents' beliefs about the causes of obesity improved, negative attitudes towards people with obesity remained high.

Two interventions had participants watching one (17‐min)^113^ or two (totaling 34‐min)^114^ anti‐stigma videos produced at Yale University. The videos employed strategies of weight controllability, empathy evoking and stereotype countering. Beliefs and stereotypes towards people with obesity improved in both trials. Attitudes improved only in the study by Swift et al.^114^ Furthermore, Swift et al.^114^ had a 6‐week follow‐up in which improved beliefs remained significant, whereas attitudes retreated back to baseline. Implicit bias did not change at any point. In both interventions, the video(s) were positively rated by both faculty and students. Still, given that attitudes did not change or started to drift with time, such brief interventions may benefit from repeated exposure, practical activities and facilitated discussions. Poustchi et al.^113^ had participants engage in discussion after the viewing but the conversation was focused on participants' experiences encountering patients with obesity, which might have contributed to the unchanged attitudes by negative value attributions. Anticipating and focusing on the narrative of the “difficult patient” has been shown to increase weight bias in medical students.^115^ Thus, not all discussions and activities around stigma are conducive to its reduction.

Two studies used lectures combined with video, articles, role‐play and reflective writing.^116^, ^117^ Both aimed to change controllability beliefs and evoke empathy and in both studies stereotyping decreased, whereas beliefs and attitudes improved according to some, but not all subscales. These changes remained significant at both 4‐week and 1‐year follow‐up. Wijayatunga et al.^117^ found that participants exhibited high implicit weight bias, which remained unchanged. Importantly, a control group, who received a traditional obesity curriculum centering exercise and diet in weight management, was more likely to show increased implicit bias at 4‐week follow‐up.^117^

Finally, two studies in kinesiology students combined lectures, addressing the multi‐factorial determinants of weight and the prevalence and effects of weight stigma, with empathy‐evoking activities and an additional field‐based service‐learning project.^118^, ^119^ In both, beliefs about controllability improved, but attitudes and stereotypes did not. Contrary to test scores, reflective writing revealed that many students had strong beliefs about personal control over weight.^118^ This somewhat higher resistance to change may relate to the field of study, as exercise science related disciplines tend to focus on physique and thinness.^120^ Rukavina et al.^119^ also measured implicit bias, which improved, but did not reach significance and thus, remained strong among the students. Again, explicit bias increased in the control group taking other classes, highlighting the possible accumulating negative effects of not addressing weight stigma in health education.^119^

There is a lack of evidence to suggest that using either single or multiple methods is better for reducing weight stigma.^121^ Most mixed methods interventions in this review were successful in changing participants' beliefs about the uncontrollable causes of obesity and in reducing blame. However, changing attitudes and implicit bias remained a challenge. Future studies that can better quantify the effects of and interactions between single approaches within a context of mixed methodology are needed. This could mean studying parallel groups with increasing number of methods or taking a stepped approach in which non‐responders receive a different type of intervention.

## 4 LIMITATIONS

Given that research regarding strategies to reduce weight stigma is currently preliminary, a narrative analysis was favored in this review to allow for discussion of emerging themes. To overcome selection bias that may characterize a traditional narrative review, a systematic database search was performed; however, there was no standardized quality assessment of the individual studies, which may reduce the strength of conclusions. Furthermore, this review focused on weight stigma reduction in HCPs, which aims to avoid future stigmatization in the healthcare setting. Although outside the scope of this study, it is important to note that many patients across the weight spectrum experience IWB,^122^ which has the potential to interfere with the patient‐provider relationship regardless of the level of bias and behavior of the professional. The role of HCPs in detecting and helping to reduce IWB in patients, especially in weight management services, deserves more attention.

## 5 CONCLUSIONS

This review looked at weight stigma reduction strategies in healthcare. Interventions involving the reduction weight bias among current and future HCPs were included. Although still more research is needed, the growing interest in weight stigma is encouraging. Around half of the studies included in this review were conducted in the past 5 years alone, providing valuable insight as we start taking broad action towards eradicating weight bias in healthcare and in society. Based on our findings, we offer three prime recommendations for stigma reduction pertaining to health education, practice and research.

First, there is a need to educate all healthcare students about the complex factors regulating body weight and address weight stigma, its prevalence, origins and impact, explicitly. The failure to address stigma among current and future HCPs upholds bias formation. Our findings show that biomedical education alone does not reduce stigma and, in most studies, control groups, when included, exhibited increased bias over time. Targeting healthcare students early on and throughout their education may be particularly beneficial because they are in the process of forming their beliefs and attitudes toward overweight and obesity, and may be more receptive to new paradigm‐shifting information. Indeed, a meta‐analysis on the malleability of weight bias by Lee et al.^88^ found that effect sizes, although not statistically significant, were considerably larger in student samples compared with professionals or trainees. While there were only four studies involving HCPs in this review, the results support this notion. Therefore, revisions to current healthcare curricula are welcomed, accounting for both the causal attribution of personal responsibility for weight and the negative value of fat. This could be achieved by ensuring there are lectures on the complexity of obesity including genetic and socioenvironmental determinants of weight regulation, as well as the science of weight‐inclusive health promotion. In this review, interventions that were based on or informed by causal information, and/or critical weight science and HAES were successful in improving explicit bias, whereas empathy‐evoking was less successful. One important distinction between, for example, the weight‐inclusive approach and the less effective empathy‐evoking approach may be in the feelings they provoke. Rather than eliciting pity by emphasizing the hardships of living with obesity, a more productive approach to reducing stigma could be to highlight common humanity and the civil right to healthcare.

Second, there is a need to move away from a solely weight‐centric approach to healthcare to a more health‐focussed approach including weight‐inclusivity. Equally important to the question of “how not to,” is the question of “how and what” we provide in healthcare services. Our findings indicate that clinical encounters are an important element in the formation of HCPs beliefs and attitudes. In several studies, negative expectations or experiences regarding patient care, compliance and outcomes contributed to weight bias retention. All healthcare facilities should be equipped with appropriately sized instruments including, but not limited to, chairs, blood pressure cuffs and gowns. Importantly, being aware of and able to use interventions that improve patients' health regardless of their weight or weight change has the potential to reduce HCPs negative experiences and stereotypes of the so called “difficult patient,” and instead promote a mutually beneficial provider‐patient relationship focussed on health. Data show that patients benefit psychologically and physically from weight‐inclusive programmes that address IWB, the psychological aspects of eating, and the social experience of living with overweight or obesity.^102^, ^123^ Addressing IWB is likely to be of greatest benefit when delivered within a weight‐inclusive health promotion programme and before commencing with behavioral weight loss because IWB is higher in those seeking weight loss when compared with the general population.^124^ It remains questionable whether and to what extent a weight‐loss goal reinforces beliefs about weight controllability and blame, and is thus in itself stigmatizing.^125^ Furthermore, working to decrease IWB within weight‐loss programmes may prove challenging because a weight‐loss goal may make improvements in IWB conditional on weight loss and maintenance. Screening for and addressing IWB in people with overweight or obesity looking to improve their eating behaviors, while also funding, designing and implementing long‐term stigma reducing interventions, may help to reduce weight bias in HCPs, as it enables continuous reinforcement and enactment on anti‐weight stigma values. Additionally, although the responsibility of reducing weight stigma in healthcare settings must fall on the provider, reducing IWB can empower patients to advocate for care they deserve.

Lastly, when conducting research on the relationship between weight, health and mortality, there is a need to ensure that researchers measure and account for the confounding and/or mediating effects of weight stigma.^24^, ^28^, ^126^ Weight stigma, as experienced and/or internalized, is largely absent from current epidemiological research, which informs medical, political, and social discourse. Preliminary research shows that a significant proportion of the relationship between obesity and health outcomes can be explained not by body weight itself, but by the negative experiences commonly shared by people with overweight and obesity. More research is needed to understand this relationship and to highlight the importance of weight stigma on health outcomes in the scientific community.

Although the ethical argument and evidence base for the need to reduce stigma in healthcare and beyond is strong, research attention needs to move towards finding rigorous empirical evidence into the specific approaches to reduce weight stigma not just in the short term but in the long‐term. Designing robust, randomized controlled trials with large population sizes and sufficient follow‐up will uphold this aim. Nevertheless, the magnitude and consequences of the issue demand precautionary action, even as evidence is still emerging. Eradicating weight stigma in society should be treated as a public health priority. This requires a whole systems approach, with co‐operation of a wide range of stakeholders among whom HCPs, educators, researchers and policymakers as well as patients play an essential role.

## CONFLICT OF INTERESTS

The authors of this manuscript certify that they comply with the ethical guidelines for authorship and publishing in the Obesity Reviews. This scoping review is based on published data and does not contain sensitive clinical study or patient data. RLB reports receiving consulting fees from Pfizer, Eli‐Lilly, Gila Therapeutics Inc., and ViiV Healthcare and consulting fees, lecture fees from Novo Nordisk and participating in clinical trials for Novo Nordisk. AB reports receiving consulting and lecture fees from Novo Nordisk outside the submitted work; and is the Vice Chair of Specialist Obesity Group of the BDA, and on the Medical Advisory Board and shareholder of Reset Health Clinics Ltd.

## AUTHOR CONTRIBUTIONS

AZK conceived the study; AZK, BT, AB, and RLB contributed to the study design and methodology; AZK was responsible for the oversight of the study; BT with support from AZK screened the selected manuscripts included in the review and prepared the draft. All authors contributed to the writing and critical revision of the manuscript and gave final approval.

## References

[1] Hatzenbuehler ML , Phelan JC , Link BG . Stigma as a fundamental cause of population health inequalities. Am J Public Health. 2013;103(5):813‐821. 23488505 10.2105/AJPH.2012.301069 PMC3682466

[2] Nancy K . Discrimination and health inequities. Int J Health Serv. 2014;44(4):643‐710. doi:10.2190/HS.44.4.b 25626224

[3] Rubino F , Puhl RM , Cummings DE , et al. Joint international consensus statement for ending stigma of obesity. Nat Med. 2020;26(4):485‐497. doi:10.1038/s41591-020-0803-x 32127716 PMC7154011

[4] Spahlholz J , Baer N , König HH , Riedel‐Heller SG , Luck‐Sikorski C . Obesity and discrimination ‐ a systematic review and meta‐analysis of observational studies. Obes Rev. 2016;17(1):43‐55. 26596238 10.1111/obr.12343

[5] Andreyeva T , Puhl RM , Brownell KD . Changes in Perceived Weight Discrimination Among Americans, 1995–1996 Through 2004–2006. Obesity. 2008;16(5):1129‐1134. 18356847 10.1038/oby.2008.35

[6] Baker P , Brookes G , Atanasova D , Flint SW . Changing frames of obesity in the UK press 2008–2017. Soc Sci Med. 2020;264:113403. 33017735 10.1016/j.socscimed.2020.113403

[7] Puhl RM , Heuer CA . Obesity stigma: Important considerations for public health. Am J Public Health. 2010;100(6):1019‐1028. doi:10.2105/AJPH.2009.159491 20075322 PMC2866597

[8] Tomiyama AJ , Carr D , Granberg EM , et al. How and why weight stigma drives the obesity ‘epidemic’ and harms health. BMC Med. 2018;16(1):1‐6. 10.1186/s12916-018-1116-5 PMC6092785 30107800

[9] Brewis AA , Wutich A , Falletta‐Cowden A , Rodriguez‐Soto I . Body Norms and Fat Stigma in Global Perspective. Curr Anthropol. 2011;52(2):269‐276.

[10] Strings S . Women (Re)making whiteness: the sexual exclusion of the fat “Black” Irish. Ethn Racial Stud. 2020;43(4):672‐689.

[11] Kyle TK . Weight shaming appears to be declining more in the USA than in the UK. AAAS. https://eurekalert.org/pub_releases/2020-09/eaft-wsa083120.php. Published 2020. Accessed 07/01/2021, 2020.

[12] Brown A , Flint SW , Batterham RL . Pervasiveness, impact and implications of weight stigma. eClinicalMedicine. 2022;47:101408. doi:10.1016/j.eclinm.2022.101408 35497065 PMC9046114

[13] Wu Y‐K , Berry DC . Impact of weight stigma on physiological and psychological health outcomes for overweight and obese adults: A systematic review. J Adv Nurs. 2018;74(5):1030‐1042. doi:10.1111/jan.13511 29171076

[14] Vadiveloo M , Mattei J . Perceived Weight Discrimination and 10‐Year Risk of Allostatic Load Among US Adults. Ann Behav Med. 2017;51(1):94‐104. doi:10.1007/s12160-016-9831-7 27553775 PMC5253095

[15] Friedman KE , Ashmore JA , Applegate KL . Recent experiences of weight‐based stigmatization in a weight loss surgery population: Psychological and behavioral correlates. Obesity. 2008;16(SUPPL. 2):S69‐S74. 18978766 10.1038/oby.2008.457

[16] Vartanian LR , Pinkus RT , Smyth JM . Experiences of weight stigma in everyday life: Implications for health motivation. Stigma Health (Washington, DC). 2018;3(2):85‐92. doi:10.1037/sah0000077

[17] McCleary‐Gaddy AT , Miller CT , Grover KW , Hodge JJ , Major B . Weight Stigma and Hypothalamic‐Pituitary‐Adrenocortical Axis Reactivity in Individuals Who Are Overweight. Ann Behav Med. 2019;53(4):392‐398. doi:10.1093/abm/kay042 29917036 PMC6426042

[18] Himmelstein MS , Incollingo Belsky AC , Janet Tomiyama A . The weight of stigma: Cortisol reactivity to manipulated weight stigma. Obesity. 2015;23(2):368‐374. 25522347 10.1002/oby.20959

[19] Miller GE , Chen E , Zhou ES . If it goes up, must it come down? Chronic stress and the hypothalamic‐pituitary‐adrenocortical axis in humans. Psychol Bull. 2007;133(1):25‐45. doi:10.1037/0033-2909.133.1.25 17201569

[20] Schvey NA , Puhl RM , Brownell KD . The stress of stigma: exploring the effect of weight stigma on cortisol reactivity. Psychosom Med. 2014;76(2):156‐162. doi:10.1097/PSY.0000000000000031 24434951

[21] Hatzenbuehler ML , McLaughlin KA . Structural stigma and hypothalamic‐pituitary‐adrenocortical axis reactivity in lesbian, gay, and bisexual young adults. Ann Behav Med. 2014;47(1):39‐47. doi:10.1007/s12160-013-9556-9 24154988 PMC3945440

[22] van der Valk ES , Savas M , van Rossum EFC . Stress and Obesity: Are There More Susceptible Individuals? Curr Obes Rep. 2018;7(2):193‐203. doi:10.1007/s13679-018-0306-y 29663153 PMC5958156

[23] Jung F , Bae Y , Kratzsch J , Riedel‐Heller S , Luck‐Sikorski C . Internalized weight bias and cortisol reactivity to social stress. Cogn Affect Behav Neurosci. 2020;20(1):49‐58. doi:10.3758/s13415-019-00750-y 31654234

[24] Daly M , Sutin AR , Robinson E . Perceived Weight Discrimination Mediates the Prospective Association Between Obesity and Physiological Dysregulation: Evidence From a Population‐Based Cohort. Psychol Sci. 2019;30(7):1030‐1039. 31158067 10.1177/0956797619849440 PMC6657150

[25] Alimoradi Z , Golboni F , Griffiths MD , Broström A , Lin C‐Y , Pakpour AH . Weight‐related stigma and psychological distress: A systematic review and meta‐analysis. Clin Nutr (Edinburgh, Scotland). 2019;39(7):2001–2013. doi:10.1016/j.clnu.2019.10.016 31732288

[26] Hayward LE , Vartanian LR , Pinkus RT . Weight Stigma Predicts Poorer Psychological Well‐Being Through Internalized Weight Bias and Maladaptive Coping Responses. Obesity. 2018;26(4):755‐761. doi:10.1002/oby.22126 29427370

[27] Puhl RM , Himmelstein MS , Pearl RL . Weight stigma as a psychosocial contributor to obesity. Am Psychol. 2020;75(2):274‐289. doi:10.1037/amp0000538 32053000

[28] Sutin AR , Stephan Y , Terracciano A . Weight Discrimination and Risk of Mortality. Psychol Sci. 2015;26(11):1803‐1811. doi:10.1177/0956797615601103 26420442 PMC4636946

[29] Remmert JE , Convertino AD , Roberts SR , Godfrey KM , Butryn ML . Stigmatizing weight experiences in health care: Associations with BMI and eating behaviours. Obes Sci Pract. 2019;5(6):555‐563. doi:10.1002/osp4.379 31890246 PMC6934430

[30] Nolan LJ , Eshleman A . Paved with good intentions: Paradoxical eating responses to weight stigma. Appetite. 2016;102:15‐24. doi:10.1016/j.appet.2016.01.027 26802721

[31] Cheng MY , Wang S‐M , Lam YY , Luk HT , Man YC , Lin C‐Y . The relationships between weight bias, perceived weight stigma, eating behavior, and psychological distress among undergraduate students in Hong Kong. J Nerv Ment Dis. 2018;206(9):705‐710. doi:10.1097/NMD.0000000000000869 30124569

[32] Araiza AM , Wellman JD . Weight stigma predicts inhibitory control and food selection in response to the salience of weight discrimination. Appetite. 2017;114:382‐390. doi:10.1016/j.appet.2017.04.009 28416329 PMC5533089

[33] Major B , Hunger JM , Bunyan DP , Miller CT . The ironic effects of weight stigma. J Exp Soc Psychol. 2014;51:74‐80. doi:10.1016/j.jesp.2013.11.009

[34] Schvey NA , Puhl RM , Brown KD . The impact of weight stigma on caloric consumption. Obesity. 2011;19(10):1957‐1962. doi:10.1038/oby.2011.204 21760636

[35] Ashmore JA , Friedman KE , Reichmann SK , Musante GJ . Weight‐based stigmatization, psychological distress, & binge eating behavior among obese treatment‐seeking adults. Eat Behav. 2008;9(2):203‐209. doi:10.1016/j.eatbeh.2007.09.006 18329599

[36] Douglas V , Varnado‐Sullivan P . Weight stigmatization, internalization, and eating disorder symptoms: The role of emotion dysregulation. Stigma Health. 2016;1(3):166‐175. doi:10.1037/sah0000029

[37] Himmelstein MS , Puhl RM , Quinn DM . Weight stigma and health: The mediating role of coping responses. Health Psychol. 2018;37(2):139‐147. doi:10.1037/hea0000575 29120192

[38] Hunger JM , Major B , Blodorn A , Miller CT . Weighed Down by Stigma: How Weight‐Based Social Identity Threat Contributes to Weight Gain and Poor Health. Soc Person Psych Compass. 2015;9(6):255‐268. doi:10.1111/spc3.12172 PMC5720363 29225670

[39] Puhl RM , Brownell KD . Confronting and coping with weight stigma: An investigation of overweight and obese adults. Obesity. 2006;14(10):1802‐1815. doi:10.1038/oby.2006.208 17062811

[40] Schwartz MB , Vartanian LR , Nosek BA , Brownell KD . The influence of one's own body weight on implicit and explicit anti‐fat bias. Obesity. 2006;14(3):440‐447. doi:10.1038/oby.2006.58 16648615

[41] Carels RA , Hlavka R , Selensky JC , Solar C , Rossi J , Caroline Miller J . A daily diary study of internalised weight bias and its psychological, eating and exercise correlates. Psychol Health. 2019;34(3):306‐320. doi:10.1080/08870446.2018.1525491 30587043

[42] O'Brien KS , Latner JD , Puhl RM , et al. The relationship between weight stigma and eating behavior is explained by weight bias internalization and psychological distress. Appetite. 2016;102:70‐76. doi:10.1016/j.appet.2016.02.032 26898319

[43] Puhl RM , Moss‐Racusin CA , Schwartz MB . Internalization of weight bias: Implications for binge eating and emotional well‐being. Obesity. 2007;15(1):19‐23. doi:10.1038/oby.2007.521 17228027

[44] Bidstrup H , Brennan L , Kaufmann L , de la Piedad Garcia X. Internalised weight stigma as a mediator of the relationship between experienced/perceived weight stigma and biopsychosocial outcomes: a systematic review. Int J Obes (Lond) 2022;46(1):1–9. doi:10.1038/s41366-021-00982-4 34628466 PMC8501332

[45] Meadows A , Higgs S . Internalised weight stigma moderates the impact of a stigmatising prime on eating in the absence of hunger in higher‐ but not lower‐weight individuals. Front Psychol. 2019;10:1022. doi:10.3389/fpsyg.2019.01022 31139111 PMC6519002

[46] Major B , Rathbone JA , Blodorn A , Hunger JM . The Countervailing Effects of Weight Stigma on Weight‐Loss Motivation and Perceived Capacity for Weight Control. Pers Soc Psychol Bull. 2020;46(9):1331‐1343. 32046597 10.1177/0146167220903184

[47] Wellman JD , Araiza AM , Newell EE , McCoy SK . Weight stigma facilitates unhealthy eating and weight gain via fear of fat. Stigma Health. 2018;3(3):186‐194. doi:10.1037/sah0000088 30221195 PMC6132275

[48] Vartanian LR , Porter AM . Weight stigma and eating behavior: A review of the literature. Appetite. 2016;102:3‐14. doi:10.1016/j.appet.2016.01.034 26829371

[49] Lee KM , Hunger JM , Tomiyama AJ . Weight stigma and health behaviors: evidence from the Eating in America Study. Int J Obes (Lond). 2021;45(7):1499‐1509. 33934109 10.1038/s41366-021-00814-5 PMC8236399

[50] Täuber S , Gausel N , Flint SW . Weight bias internalization: The maladaptive effects of moral condemnation on intrinsic motivation. Front Psychol. 2018;9:1836. doi:10.3389/fpsyg.2018.01836 30319517 PMC6170635

[51] Lin CY , Strong C , Latner JD , Lin YC , Tsai MC , Cheung P . Mediated effects of eating disturbances in the association of perceived weight stigma and emotional distress. Eat Weight Disord. 2020;25(2):509‐518. 30697663 10.1007/s40519-019-00641-8

[52] Tomiyama A . Weight stigma is stressful. A review of evidence for the Cyclic Obesity/Weight‐Based Stigma model. Appetite. 2014;82:8‐15. 24997407 10.1016/j.appet.2014.06.108

[53] Puhl RM , Lessard LM , Himmelstein MS , Foster GD . The roles of experienced and internalized weight stigma in healthcare experiences: Perspectives of adults engaged in weight management across six countries. PLoS ONE. 2021;16(6):e0251566. doi:10.1371/journal.pone.0251566 34061867 PMC8168902

[54] Hansson LM , Näslund E , Rasmussen F . Perceived discrimination among men and women with normal weight and obesity. A population‐based study from Sweden. Scand J Public Health. 2010;38(6):587‐596. doi:10.1177/1403494810372266 20529966

[55] Puhl RM , Heuer CA . The Stigma of Obesity: A Review and Update. Obesity. 2009;17(5):941‐964. doi:10.1038/oby.2008.636 19165161

[56] Miller DP , Spangler JG , Vitolins MZ , et al. Are medical students aware of their anti‐obesity bias? Acad Med. 2013;88(7):978‐982. 23702519 10.1097/ACM.0b013e318294f817 PMC3930920

[57] George TP , DeCristofaro C , Murphy PF . Unconscious Weight Bias Among Nursing Students: A Descriptive Study. Healthcare (Basel). 2019;7(3):106. doi:10.3390/healthcare7030106 PMC6787661 31547359

[58] Phelan SM , Burgess DJ , Yeazel MW , Hellerstedt WL , Griffin JM , van Ryn M . Impact of weight bias and stigma on quality of care and outcomes for patients with obesity. Obes Rev. 2015;16(4):319‐326. doi:10.1111/obr.12266 25752756 PMC4381543

[59] Fitzgerald C , Hurst S . Implicit bias in healthcare professionals: A systematic review. BMC Med Ethics. 2017;18(1):19. doi:10.1186/s12910-017-0179-8 28249596 PMC5333436

[60] Nyblade L , Stockton MA , Giger K , et al. Stigma in health facilities: Why it matters and how we can change it. BMC Med. 2019;17(1):25. doi:10.1186/s12916-019-1256-2 30764806 PMC6376713

[61] Alberga AS , Edache IY , Forhan M , Russell‐Mayhew S . Weight bias and health care utilization: A scoping review. Prim Health Care Res Dev. 2019;20:e116. 32800008 10.1017/S1463423619000227 PMC6650789

[62] Hayward LE , Neang S , Ma S , Vartanian LR . Discussing weight with patients with overweight: Supportive (not stigmatizing) conversations increase compliance intentions and health motivation. Stigma Health (Washington, DC). 2020;5(1):53‐68.

[63] Fatima Cody S , Erica DJ , Mechelle DC , Rebecca LE , Lee MK . The Role of Obesity Training in Medical School and Residency on Bariatric Surgery Knowledge in Primary Care Physicians. Int J Family Med. 2015;2015:841249‐841248. 26339506 10.1155/2015/841249 PMC4539067

[64] Vitolins MZ , Crandall S , Miller D , Ip E , Marion G , Spangler JG . Obesity Educational Interventions in U.S. Medical Schools: A Systematic Review and Identified Gaps. Teach Learn Med. 2012;24(3):267‐272. doi:10.1080/10401334.2012.692286 22775792 PMC3811015

[65] Barra M , Singh Hernandez SS . Too big to be seen: Weight‐based discrimination among nursing students. Nurs Forum. 2018;53(4):529‐534. doi:10.1111/nuf.12282 29968365

[66] Gayer GG , Weiss J , Clearfield M . Fundamentals for an Osteopathic obesity designed study: The effects of education on osteopathic medical students' attitudes regarding obesity. J am Osteopathic Assoc. 2017;117(8):495‐502. doi:10.7556/jaoa.2017.099 28759091

[67] Geller G , Watkins PA . Addressing medical students' negative bias toward patients with obesity through ethics education. AMA J Ethics. 2018;20(10):948‐959. doi:10.1001/amajethics.2018.948 30346923

[68] Jones CA , Forhan M . Addressing weight bias and stigma of obesity amongst physiotherapists. Physiother Theory Pract. 2021;37(7):808‐816. 31362578 10.1080/09593985.2019.1648623

[69] Nickel F , Tapking C , Benner L , et al. Video Teaching Leads to Improved Attitudes Towards Obesity‐a Randomized Study with 949 Participants. Obes Surg. 2019;29(7):2078‐2086. doi:10.1007/s11695-019-03804-9 30838534

[70] Zitek EM , Hebl MR . The role of social norm clarity in the influenced expression of prejudice over time. J Exp Soc Psychol. 2007;43(6):867‐876.

[71] Batras D , Duff C , Smith BJ . Organizational change theory: implications for health promotion practice. Health Promot Int. 2016;31(1):231‐241. 25398838 10.1093/heapro/dau098

[72] Wynn T , Islam N , Thompson C , Myint KS . The effect of knowledge on healthcare professionals' perceptions of obesity. Obes Med. 2018;11:20‐24. doi:10.1016/j.obmed.2018.06.006

[73] O'Brien KS , Hunter JA , Banks M . Implicit anti‐fat bias in physical educators: physical attributes, ideology and socialization. Int J Obes (Lond). 2007;31(2):308‐314. doi:10.1038/sj.ijo.0803398 16733526

[74] Rathbone JA , Cruwys T , Jetten J , Barlow FK . When stigma is the norm: How weight and social norms influence the healthcare we receive. J Appl Soc Psychol. 2020. doi:10.1111/JASP.12689

[75] Phelan SM , Puhl RM , Burke SE , et al. The mixed impact of medical school on medical students' implicit and explicit weight bias. Med Educ. 2015;49(10):983‐992. doi:10.1111/medu.12770 26383070 PMC4755318

[76] Bergen M , Mollen D . Teaching Sizeism: Integrating Size into Multicultural Education and Clinical Training. Women Therapy. 2019;42(1–2):164‐180.

[77] Teachman BA , Gapinski KD , Brownell KD , Rawlins M , Jeyaram S . Demonstrations of implicit anti‐fat bias: the impact of providing causal information and evoking empathy. Health Psychol. 2003;22(1):68‐78. doi:10.1037/0278-6133.22.1.68 12558204

[78] Puhl RM , Latner JD , O'Brien K , Luedicke J , Danielsdottir S , Forhan M . A multinational examination of weight bias: predictors of anti‐fat attitudes across four countries. Int J Obes (Lond). 2015;39(7):1166‐1173. doi:10.1038/ijo.2015.32 25809827

[79] Hilbert A . Weight stigma reduction and genetic determinism. PLoS ONE. 2016;11(9):e0162993. doi:10.1371/journal.pone.0162993 27631384 PMC5025056

[80] Brochu PM . Testing the effectiveness of a weight bias educational intervention among clinical psychology trainees. J Appl Soc Psychol. 2020. doi:10.1111/jasp.12653

[81] Diedrichs PC , Barlow FK . How to lose weight bias fast! Evaluating a brief anti‐weight bias intervention. Br J Health Psychol. 2011;16(4):846‐861. 21988068 10.1111/j.2044-8287.2011.02022.x

[82] O'Brien KS , Puhl RM , Latner JD , Mir AS , Hunter JA . Reducing anti‐fat prejudice in preservice health students: A randomized trial. Obesity. 2010;18(11):2138‐2144. doi:10.1038/oby.2010.79 20395952

[83] Persky S , Eccleston CP . Impact of genetic causal information on medical students' clinical encounters with an obese virtual patient: Health promotion and social stigma. Ann Behav Med. 2011;41(3):363‐372. doi:10.1007/s12160-010-9242-0 21136226 PMC3098938

[84] Crandall CS , D'Anello S , Sakalli N , Lazarus E , Nejtardt GW , Feather NT . An Attribution‐Value Model of Prejudice: Anti‐Fat Attitudes in Six Nations. Pers Soc Psychol Bull. 2016;27(1):30‐37. doi:10.1177/0146167201271003

[85] Daníelsdóttir S , O'Brien KS , Ciao A . Anti‐Fat Prejudice Reduction: A Review of Published Studies. Obes Facts. 2010;3(1):47‐58. doi:10.1159/000277067 20215795 PMC6452150

[86] Derksen F , Bensing J , Lagro‐Janssen A . Effectiveness of empathy in general practice: a systematic review. Br J Gen Pract. 2013;63(606):e76‐e84. 23336477 10.3399/bjgp13X660814 PMC3529296

[87] Moudatsou M , Stavropoulou A , Philalithis A , Koukouli S . The Role of Empathy in Health and Social Care Professionals. Healthcare (Basel). 2020;8(1):26. doi:10.3390/healthcare8010026 PMC7151200 32019104

[88] Lee M , Ata RN , Brannick MT . Malleability of weight‐biased attitudes and beliefs: A meta‐analysis of weight bias reduction interventions. Body Image. 2014;11(3):251‐259. doi:10.1016/j.bodyim.2014.03.003 24958660

[89] Cotugna N , Mallick A . Following a calorie‐restricted diet may help in reducing healthcare students' Fat‐Phobia. J Community Health. 2010;35(3):321‐324. doi:10.1007/s10900-010-9226-9 20130971

[90] Harris MB , Walters LC , Waschull S . Altering Attitudes and Knowledge about Obesity. J Soc Psychol. 1991;131(6):881‐884. 1816471 10.1080/00224545.1991.9924675

[91] Hunter J , Rawlings‐Anderson K , Lindsay T , Bowden T , Aitken LM . Exploring student nurses' attitudes towards those who are obese and whether these attitudes change following a simulated activity. Nurse Educ Today. 2018;65:225‐231. doi:10.1016/j.nedt.2018.03.013 29604606

[92] Kushner RF , Zeiss DM , Feinglass JM , Yelen M . An obesity educational intervention for medical students addressing weight bias and communication skills using standardized patients. BMC Med Educ. 2014;14(1):53. doi:10.1186/1472-6920-14-53 24636594 PMC3995306

[93] Matharu K , Shapiro JF , Hammer RR , Kravitz RL , Wilson MD , Fitzgerald FT . Reducing obesity prejudice in medical education. Educ Health: Change Learn Pract. 2014;27(3):231‐237. doi:10.4103/1357-6283.152176 25758385

[94] Molloy MA , Sabol VK , Silva SG , Guimond ME . Using Trigger Films as a Bariatric Sensitivity Intervention: Improving Nursing Students' Attitudes and Beliefs About Caring for Obese Patients. Nurse Educ. 2016;41(1):19‐24. doi:10.1097/NNE.0000000000000225 26448157

[95] Meadows A , Daníelsdóttir S , Calogero R , O'Reilly C . Why fat suits do not advance the scientific study of weight stigma. Obesity. 2017;25(2):275‐275. doi:10.1002/oby.21742 28078830

[96] Gloor JL , Puhl RM . Empathy and perspective‐taking: Examination and comparison of strategies to reduce weight stigma. Stigma Health (Washington, DC). 2016;1(4):269‐279.

[97] Murakami JM , Latner JD . Weight acceptance versus body dissatisfaction: Effects on stigma, perceived self‐esteem, and perceived psychopathology. Eat Behav. 2015;19:163‐167. doi:10.1016/j.eatbeh.2015.09.010 26418164

[98] Fildes A , Charlton J , Rudisill C , Littlejohns P , Prevost AT , Gulliford MC . Probability of an Obese Person Attaining Normal Body Weight: Cohort Study Using Electronic Health Records. Am J Publ Health (1971). 2015;105(9):e54‐e59. 10.2105/AJPH.2015.302773 PMC4539812 26180980

[99] Nordmo M , Danielsen YS , Nordmo M . The challenge of keeping it off, a descriptive systematic review of high‐quality, follow‐up studies of obesity treatments. Obes Rev. 2019;21(1):e12949. 31675146 10.1111/obr.12949

[100] Montani JP , Schutz Y , Dulloo AG . Dieting and weight cycling as risk factors for cardiometabolic diseases: who is really at risk? Obes Rev. 2015;16(S1):7‐18. doi:10.1111/obr.12251 25614199

[101] Schaumberg K , Anderson D . Dietary restraint and weight loss as risk factors for eating pathology. Eat Behav. 2016;23:97‐103. doi:10.1016/j.eatbeh.2016.08.009 27611582

[102] Bacon L , Aphramor L . Weight science: evaluating the evidence for a paradigm shift. Nutr J. 2011;10:9. doi:10.1186/1475-2891-10-9 21261939 PMC3041737

[103] Hunger JM , Smith JP , Tomiyama AJ . An Evidence‐Based Rationale for Adopting Weight‐Inclusive Health Policy. Soc Issues Policy Rev. 2020;14(1):73‐107.

[104] Tylka TL , Annunziato RA , Burgard D , et al. The weight‐inclusive versus weight‐normative approach to health: evaluating the evidence for prioritizing well‐being over weight loss. J Obes. 2014;2014:983495. 25147734 10.1155/2014/983495 PMC4132299

[105] McVey GL , Walker KS , Beyers J , Harrison HL , Simkins SW , Russell‐Mayhew S . Integrating weight bias awareness and mental health promotion into obesity prevention delivery: a public health pilot study. Prev Chronic Dis. 2013;10:E46. 23557637 10.5888/pcd10.120185 PMC3617990

[106] Werkhoven T . Designing, implementing and evaluating an educational intervention targeting weight bias and fat stereotyping. J Health Psychol. 2020;26(12):2084‐2097. doi:10.1177/1359105319901310 31960717

[107] Patterson D , Buse K , Magnusson R , Toebes B . Identifying a human rights‐based approach to obesity for States and civil society. Obes Rev. 2019;20(Suppl 2):45‐56. doi:10.1111/obr.12873 31297936

[108] Calogero RM , Tylka TL , Mensinger JL , Meadows A , Daníelsdóttir S . Recognizing the Fundamental Right to be Fat: A Weight‐Inclusive Approach to Size Acceptance and Healing From Sizeism. Women Therapy. 2019;42(1–2):22‐44.

[109] Barry VW , Baruth M , Beets MW , Durstine JL , Liu J , Blair SN . Fitness vs. Fatness on All‐Cause Mortality: A Meta‐Analysis. Prog Cardiovasc Dis. 2014;56(4):382‐390. doi:10.1016/j.pcad.2013.09.002 24438729

[110] Carlsson AC , Wändell PE , Gigante B , Leander K , Hellenius M‐L , de Faire U . Seven modifiable lifestyle factors predict reduced risk for ischemic cardiovascular disease and all‐cause mortality regardless of body mass index: A cohort study. Int J Cardiol. 2013;168(2):946‐952. doi:10.1016/j.ijcard.2012.10.045 23181992

[111] Falker AJ , Sledge JA . Utilizing a bariatric sensitivity educational module to decrease bariatric stigmatization by healthcare professionals. Bariatric Nurs Surg Patient Care. 2011;6(2):73‐78. doi:10.1089/bar.2011.9974

[112] Luig T , Wicklum S , Heatherington M , et al. Improving obesity management training in family medicine: Multi‐methods evaluation of the 5AsT‐MD pilot course. BMC Med Educ. 2020;20(1):5. doi:10.1186/s12909-019-1908-0 31910854 PMC6947955

[113] Poustchi Y , Saks NS , Piasecki AK , Hahn KA , Ferrante JM . Brief intervention effective in reducing weight bias in medical students. Fam Med. 2013;45(5):345‐348. 23681687 PMC3791507

[114] Swift JA , Tischler V , Markham S , et al. Are anti‐stigma films a useful strategy for reducing weight bias among trainee healthcare professionals? Results of a pilot randomized control trial. Obes Facts. 2013;6(1):91‐102. doi:10.1159/000348714 23466551 PMC5644731

[115] Goss AL , Rethy L , Pearl RL , De Lisser HM . The “difficult” cadaver: weight bias in the gross anatomy lab. Med Educ Online. 2020;25(1):1742966. doi:10.1080/10872981.2020.1742966 32182202 PMC7144266

[116] Wiese HJ , Wilson JF , Jones RA , Neises M . Obesity stigma reduction in medical students. Int J Obes Relat Metab Disord. 1992;16(11):859‐868. 1337340

[117] Wijayatunga NN , Kim Y , Butsch WS , Dhurandhar EJ . The effects of a teaching intervention on weight bias among kinesiology undergraduate students. Int J Obes (Lond). 2019;43(11):2273‐2281. doi:10.1038/s41366-019-0325-0 30755698

[118] Rukavina PB , Li W , Rowell MB . A service learning based intervention to change attitudes toward obese individuals in kinesiology pre‐professionals. Soc Psychol Educ. 2008;11(1):95‐112. doi:10.1007/s11218-007-9039-6

[119] Rukavina PB , Li W , Shen B , Sun H . A service learning based project to change implicit and explicit bias toward obese individuals in kinesiology pre‐professionals. Obes Facts. 2010;3(2):117‐126. doi:10.1159/000302794 20484945 PMC6452103

[120] Harris J . Physical education teacher education students' knowledge, perceptions and experiences of promoting healthy, active lifestyles in secondary schools. Physical Education and Sport Pedagogy: School physical education curricula for future generations: Global neo‐liberalism? Global Lessons? 2014;19(5):466‐480.

[121] Alberga AS , Pickering BJ , Alix Hayden K , et al. Weight bias reduction in health professionals: a systematic review: Weight bias reduction in health professionals. Clin Obes. 2016;6(3):175‐188. doi:10.1111/cob.12147 27166133

[122] Romano KA , Heron KE , Henson JM . Examining associations among weight stigma, weight bias internalization, body dissatisfaction, and eating disorder symptoms: Does weight status matter? Body Image. 2021;37:38‐49. 33556915 10.1016/j.bodyim.2021.01.006 PMC8187267

[123] Schaefer JT , Magnuson AB . A review of interventions that promote eating by internal cues. J Acad Nutr Diet. 2014;114(5):734‐760. doi:10.1016/j.jand.2013.12.024 24631111

[124] Pearl RL , Himmelstein MS , Puhl RM , Wadden TA , Wojtanowski AC , Foster GD . Weight bias internalization in a commercial weight management sample: prevalence and correlates. Obes Sci Pract. 2019;5(4):342‐353. doi:10.1002/osp4.354 31452919 PMC6700514

[125] Reinka M , Quinn D , Puhl R . Examining the relationship between weight controllability beliefs and eating behaviors: The role of internalized weight stigma and BMI. Appetite. 2021;164:105257. doi:10.1016/j.appet.2021.105257 33864861

[126] Sutin AR , Terracciano A . Perceived Weight Discrimination and Obesity. PLoS ONE. 2013;8(7):e70048. 23894586 10.1371/journal.pone.0070048 PMC3722198

